# Evaluation of the Analytical Performance of the STANDARD Q COVID‐19 Antigenic Test in Suspected COVID‐19 Cases in Bobo‐Dioulasso, Burkina Faso

**DOI:** 10.1155/bmri/9435057

**Published:** 2026-02-05

**Authors:** Yacouba Sawadogo, Aicha Ilboudo, Herman Karim Sombie, Jessica Julie Chantal Samba, Noutin Fernand Michodigni, Cheick Ahmed Ouattara, Abdoul Aziz Belem, Abdoul-Salam Ouédraogo

**Affiliations:** ^1^ Emerging and Re-Emerging Pathogens Laboratory, Sourô Sanou University Hospital Center, Bobo-Dioulasso, Burkina Faso; ^2^ Bacteriology–Virology Department, Sourô Sanou University Hospital Center, Bobo-Dioulasso, Burkina Faso; ^3^ Laboratory of Molecular Biology and Genetics (LABIOGENE), Joseph Ki-Zerbo University, Ouagadougou, Burkina Faso; ^4^ Department of Higher Education, Research, and Innovation, Pietro Annigoni Biomolecular Research Center (CERBA), Ouagadougou, Burkina Faso; ^5^ Department of Information, Research, Epidemiology, and Planning, Sourô Sanou University Hospital Center, Bobo-Dioulasso, Burkina Faso; ^6^ Faculty of Medicine and Pharmacy, Nazi Boni University, Bobo-Dioulasso, Burkina Faso, univ-bobo.bf

**Keywords:** accuracy, antigen test, Cohen′s kappa, RT-PCR, SARS-CoV-2, sensitivity, specificity

## Abstract

**Background:**

The use of alternative technique such as rapid antigen diagnostic tests (Ag‐RDTs) is needed in countries with limited resources for fast tracking of COVID‐19 cases. This study evaluated the performance of the STANDARD Q COVID‐19 antigen test compared with reverse transcriptase polymerase chain reaction (RT‐PCR) among suspected COVID‐19 cases using nasopharyngeal and/or oropharyngeal swabs in Bobo‐Dioulasso, Burkina Faso.

**Methods:**

A cross‐sectional study was conducted from April to September 2021. The respiratory secretions of suspected COVID‐19 cases were collected at the Virology Laboratory of Sourô Sanou University Hospital in Bobo‐Dioulasso. The respiratory secretions consisted of nasopharyngeal or combined nasopharyngeal/oropharyngeal swabs. Each sample was analyzed using the STANDARD Q COVID‐19 Ag test and RT‐PCR on the Abbott m2000 RealTime system.

**Results:**

A total of 152 respiratory specimens were collected from suspected COVID‐19 cases and processed in this study. The sensitivity and specificity of the Ag‐RDT were 26.32% (9.15–57.20) and 98.65% (92.7–99.9), respectively, in the suspected cases with nasopharyngeal swabs from suspected cases, while a sensitivity of 25.0% (3.2–65.1) and a specificity of 98.65% (92.7–99.9) were shown with combined nasopharyngeal/oropharyngeal swabs from suspected cases. The accuracy and Cohen′s kappa of the test were 83.87% and 0.36 in suspected cases with nasopharyngeal swabs from suspected cases, while they were 83.37% and 0.32 in oropharyngeal swabs. In our study, Ag‐RDT was not sensitive on the specimens of suspected COVID‐19 cases with Ct values ≥ 20, whereas its sensitivity increased to 41.18% (18.4–67.1) on specimens with Ct < 20.

**Conclusion:**

The study findings have shown that the Ag‐RDT is useful in suspected COVID‐19 cases. However, the sensitivity of the STANDARD Q COVID‐19 Ag test was low with minimal agreement in comparison to RT‐PCR on the Abbott m2000rt system.

## 1. Introduction

Since late 2019, a new coronavirus outbreak has plagued the world, infecting over 10 million people and resulting in more than 500,000 deaths globally [1]. Coronavirus Disease 2019 (COVID‐19) is caused by SARS‐CoV‐2 (severe acute respiratory syndrome Coronavirus 2), a newly emerged coronavirus that was first identified in Wuhan, China, in December 2019 [2].

Globally, the number of confirmed cases was over 600 million according to the World Health Organization (WHO) in January 2023 [3]. Burkina Faso has been responding to the COVID‐19 pandemic since March 9, 2020, and recorded in 2022 more than 20,000 confirmed cases including 396 deaths in the same period.

Regarding this situation, the Ministry of Health, in collaboration with its technical and financial partners, has developed a response plan by involving all actors of the health system for screening and management of suspected COVID‐19 cases. The gold standard technique for the diagnosis of COVID‐19 is RT‐PCR (reverse transcriptase polymerase chain reaction) which allows the detection of viral RNA in a nasopharyngeal or combined nasopharyngeal/oropharyngeal swabs [4]. In low‐income countries, access to this type of test is limited, and efforts have been made to develop an alternative diagnostic test such as rapid antigen diagnostic tests (Ag‐RDTs) which enable the detection of SARS‐CoV‐2‐specific antigens. However, Ag‐RDTs are designed to directly detect SARS‐CoV‐2 proteins produced during virus replication in respiratory secretions which are considered less sensitive than molecular tests [5]. These antigenic tests play a crucial role in the diagnosis or screening of COVID‐19 due to their ability to detect active COVID‐19 cases. Thus, both the serological and molecular tests are used for COVID‐19 diagnosis at the national level. A few evaluations have been carried out in Ouagadougou on rapid Ag‐RDTs [6–8]. However, to our knowledge, the analytical performance of Ag‐RDT is not evaluated on potential respiratory secretions for COVID‐19 diagnosis in Bobo‐Dioulasso, Burkina Faso. It is within this framework that this study was conducted to evaluate the analytical performance of the STANDARD Q COVID‐19 antigen test in comparison to RT‐PCR with different respiratory secretions from suspected COVID‐19 cases in Bobo‐Dioulasso, Burkina Faso.

## 2. Methods

### 2.1. Type, Period, and Sampling Area of the Study

This was a descriptive cross‐sectional study conducted from April to September 2021. It was carried out at the Sourô Sanou University Teaching Hospital (SS‐UTH) in Bobo‐Dioulasso. The laboratory analyses were performed at the Bacteriology–Virology Laboratory of the SS‐UTH.

### 2.2. Study Population

The study population was composed of suspected cases of COVID‐19 living in the city of Bobo‐Dioulasso, whose samples were sent to the Bacteriology–Virology Laboratory of SS‐UTH. Only suspected COVID‐19 patients who were tested using a STANDARD Q COVID‐19 kit test during the study period were included in this study. Patients who did not receive a PCR test on the Abbott m2000 system were excluded.

This was an exhaustive sampling of nasopharyngeal or oropharyngeal swabs from suspected COVID‐19 cases who had received a STANDARD Q COVID‐19 test.

### 2.3. Sampling and Data Collection

Suspected COVID‐19 cases received for screening of COVID‐19 at the SS‐UTH were included in this study. Samples consisted of nasopharyngeal and/or oropharyngeal swabs stored in 3 mL viral transport media (VTM). All patients who were screened through RT‐PCR for the diagnosis of COVID‐19 were included in this study, regardless of the nature of their specimens. Sociodemographic and clinical data were collected from COVID‐19 case report forms. Study variables were sex, age, district of residence, occupation, patient status, nature of specimen, clinical signs at investigation, and results of antigenic test (STANDARD Q COVID‐19 Ag) and RT‐PCR assay (Abbott RealTime SARS‐CoV‐2) tests.

### 2.4. Realization of Ag‐RDT and RT‐PCR Assays on Abbott m2000 RealTime System

The diagnosis of COVID‐19 was performed with the STANDARD Q COVID‐19 Ag test (SD BIOSENSOR, Yeongtong‐gu, Suwon‐si, South Korea), which is an immunochromatographic test used for qualitative detection of SARS‐CoV‐2′s core antigens in human nasopharynx (SARS‐CoV‐2 nucleocapsid protein antigen). Briefly, 350 *μ*L volume of the sample was mixed with the extraction buffer and added to the sample well (three drops) of the cartridges. The duration for interpretation of the test was 15–30 min. For PCR testing, the Abbott m2000 Real Time system (Abbott Molecular, Taipei, Taiwan) was used, combining automatic extraction of SARS‐CoV‐2 RNA with a sample volume of 1000 *μ*L. To detect the presence of SARS‐CoV‐2 RNA by RT‐PCR, the Abbott RealTime SARS‐CoV‐2 kit was used. An internal control was used throughout the process. The time required for a series of tests is approximately 6 h. Results were considered positive when the cycle threshold (Ct) values of the N and RdRp genes were less than 31.5.

### 2.5. Data Analysis

Data analysis was performed with Microsoft Excel 2016 and SPSS (Statistical Package for the Social Sciences) Version 20.0. Quantitative variables were expressed as medians and qualitative variables as frequencies and proportions. Sensitivity (Se), specificity (Sp), positive predictive value (PPV), negative predictive value (NPV), accuracy, kappa coefficient (kappa), and their 95% CI were calculated to determine the predictive validity of the STANDARD Q COVID‐19 Ag test and the level of its agreement with the RT‐qPCR test on the Abbott m2000 RealTime [9]. Results of the STANDARD Q COVID‐19 antigen test were compared to those of RT‐qPCR, which was considered the gold standard test for this evaluation. When the accuracy value of the test was above 0.70, the test was considered useful, while a value above 0.90 was considered to have high diagnostic value [10]. Cohen′s kappa values above 0.9 and between 0 and 0.20 were defined as perfect and no agreement, respectively (Table 1) [11].

**Table 1:** Interpretation of Cohen′s kappa.

**Value of kappa**	**Level of agreement**	**% of data that are reliable**
0–0.20	None	0%–4%
0.21–0.39	Minimal	4%–15%
0.40–0.59	Weak	15%–35%
0.60–0.79	Moderate	35%–63%
0.80–0.90	Strong	64%–81%
Above 0.90	Almost perfect	82%–100%

#### 2.5.1. Ethical Considerations

An authorization letter was issued by the General Director of the SS‐UTH for data collection (No. 2021‐03‐050‐MSHP/SG/CHUSS/DG/DL/SBV/LaPathE/March 2021). Anonymity and confidentiality with respect to the data collected from the patients were observed throughout and after this study. Signed and written informed consent was given by each participant.

## 3. Results

### 3.1. General Characteristics of the Study Population

A total of 152 suspected COVID‐19 cases were included in this study. The majority of suspected cases were female with a proportion of 54% and a sex ratio of 1.17 (F/M) (Table 2). The age of the participants ranged from 16 to 95 years old with a mean of 50.78 ± 15.55 years. The most represented age group was 35–45 years, followed by 45–55 years with frequencies of 21.05% and 15.79%, respectively. The most represented professional category was civil servants accounting for 42.11%, followed by housemaids and retired persons with proportions of 21.71% and 13.82%, respectively. Suspected cases with cough represented 15% of the study population, followed by those with asthenia. Shortness of breath, fever, and/or shivering accounted for 12%. Nasopharyngeal swabs represented 61.2% of the samples.

**Table 2:** General characteristics of the study population.

**Parameters**		**Number of participants (** **n** = 152**)**	**Proportion (%) 100**
Gender	Female	82	53.95
	Male	70	46.05

Age	≤ 25	13	8.55
	25–35	19	12.50
	35–45	32	21.05
	45–55	24	15.79
	55–65	23	15.13
	65–75	24	15.79
	75–85	13	8.55
	85–95	4	2.63

Profession	Civil servants	64	42.11
	Housemaids	33	21.71
	Traders	8	5.26
	Students/pupil	9	5.92
	Retirees	21	13.82
	Other	12	7.90
	Missing data	5	3.28

Clinical signs	Fever/shivering	84	12.00
	Shortness of breath	81	12.00
	Coughing	102	15.00
	Asthenia	83	12.00
	Diarrhea	15	2.00
	Nausea/vomiting	31	4.00
	Pain	75	11.00
	Sore throat	27	4.00
	Headaches	72	10.00
	Nasal discharge	34	5.00
	Irritability/mental confusion	6	1.00
	Anosmia	34	5.00
	Ageusia	26	4.00
	Other symptoms	20	3.00

Nature of the specimens	Nasopharyngeal	93	61.18
	Nasal/oropharyngeal	53	34.87
	Oropharyngeal	6	3.95

### 3.2. Performance of the STANDARD Q COVID‐19 Test According to the Type of Sample

This study showed that the Se and the Sp of the rapid diagnostic test were 25.93 (13.1–44.7) and 98.40 (94.3–99.6), respectively, in all the suspected COVID‐19 cases, in comparison to RT‐PCR with an accuracy and Cohen′s kappa of 85.53 and 0.35, respectively. The Se and Sp of the test were 26.32% (9.15–57.20) and 98.65% (92.7–99.9), respectively, with nasopharyngeal swabs from the suspected cases (Table 3), while an Se of 25.0% (3.2–65.1) and an Sp of 98.65% (92.7–99.9) were shown with oropharyngeal swabs from the suspected cases with oropharyngeal swabs. The accuracy and Cohen′s kappa of the test were 83.87% and 0.36 in suspected cases with nasopharyngeal swabs, while they were 83.37% and 0.32 in suspected cases with oropharyngeal swabs. The performance of the STANDARD Q is shown in Table 3.

**Table 3:** Performance of the STANDARD Q COVID‐19 test according to the type of specimens.

**Parameters**	**All suspected cases (** **n** = 152**) (100%)**	**Nasopharyngeal (** **n** = 93**) (61.18%)**	**Combined nasopharyngeal/oropharyngeal (** **n** = 53**) (34.87%)**
Sensitivity [95% CI]^a^	25.93 [13.1–44.7]	26.32 [9.2–51.2]	25.00 [3.2–65.1]
Specificity [95% CI]	98.40 [94.3–99.6]	98.65 [92.7–99.9]	97.78 [88.2–99.9]
PPV^b^ [95% CI]	77.78 [44.7–94.0]	83.33 [35.9–99.6]	66.67 [9.4–99.9]
NPV^c^ [95% CI]	86.01 [79.4–90.8]	83.91 [74.5–90.9]	66.67 [9.4–99.2]
Accuracy (%)	85.53	83.87	86.79
Kappa	0.35	0.36	0.32

^a^Confidence interval at 95%.

^b^Positive predictive value.

^c^Negative predictive value.

### 3.3. Performance of the STANDARD Q COVID‐19 Test According to Ct Value of Abbott m2000 System

Out of the 27 positive results on the Abbott m2000 SARS‐CoV‐2 system, seven of them were positive using the STANDARD Q COVID‐19 kit.

Positive samples with Ct values < 20 on the Abbott m2000 system predominated in this study, and the antigenic test showed an Se of 41.18% (18.4–67.1) in all suspected COVID‐19 cases. Positive specimens with Ct values ≥ 20 on the Abbott system were not detected through antigenic test using the STANDARD Q COVID‐19 kit.

### 3.4. Performance of the STANDARD Q COVID‐19 Test According to Clinical Signs

The Se of the STANDARD Q COVID‐19 test according to clinical signs was in decreasing order 81.48% (63.3–91.82), 66.67% (47.82–81.36), 62.96% (44.23–78.47), and 62.96% (44.23–78.47) in confirmed COVID‐19 cases with cough, asthenia, fever/shivering, shortness of breath, and headache, respectively (Table 4).

**Table 4:** Diagnostic performance of the STANDARD Q COVID‐19 test according to clinical signs.

**Clinical sign (%)**	**Sensitivity [95% CI]**	**Specificity [95% CI]**
Fever/shivering (12)^a^	62.96 [44.2–78.5]	46.40 [37.6–55.1]
Shortness of breath (12)^a^	62.96 [44.2–78.5]	48.80 [40.2–57.5]
Cough (15)^a^	81.48 [63.3–91.8]	36.00 [28.1–44.7]
General weakness (12)^a^	66.67 [47.8–81.4]	48.00 [39.4–56.7]
Diarrhea (2)	7.41 [2.1–23.4]	89.60 [83.0–93.8]
Nausea/vomiting (4)	18.52 [8.2–36.7]	77.97 [69.7–84.5]
Pain (11)^a^	51.85 [33.9–69.3]	51.20 [42.5–59.8]
Sore throat (4)	14.81 [5.9–32.5]	81.60 [73.9–87.4]
Headache (10)^a^	62.96 [44.2–78.5]	56.00 [47.2–64.4]
Nasal discharge (5)	22.22 [10.6–40.8]	76.80 [68.7–83.3]
Irritability/mental confusion (1)	3.70 [0.1–18.3]	96.00 [90.9–98.3]
Ageusia (5)	33.33 [18.6–52.2]	80.00 [72.1–86.1]
Anosmia (4)	29.63 [15.8–48.5]	85.60 [78.4–90.7]
Other symptoms (3)	7.41 [2.1–23.4]	85.60 [78.4–90.7]

^a^Group with sensitivity higher than 50%.

## 4. Discussion

The objective of this study was to evaluate the diagnostic performance of the STANDARD Q COVID‐19 antigen test versus the RT‐PCR test with different respiratory secretions from suspected COVID‐19 in Bobo‐Dioulasso, Burkina Faso. Regarding the sociodemographic status of the suspected COVID‐19 cases, the female gender was the most predominant in this study population and accounted for 54%. This female predominance in COVID‐19 cases was reported by other authors such as Samaké et al. in Mali in 2020 [12]. This could be a reflection of the structure of the general population of Burkina Faso. Indeed, women represented 51.7% of the population of Burkina Faso in the fifth general census of the human population in 2021, according to the National Institute of Statistics and Demography (INSD). The mean age of suspected COVID‐19 cases was 50.78 ± 15.55 years in this study. This finding correlates with the study conducted in Ouagadougou by Sawadogo et al. in 2020 who reported a mean age of 53 ± 18 years [13]. Civil servants were the majority in our study population and represented 42.11% of the population. This finding is similar to the finding of Donamou et al. in 2021 in Guinea who showed a predominance of civil servants in their study population (68%). These results could be explained by the multiple trips that the civil servants make in line with their duties [14].

The analytical performance of the Ag‐RDT depends on various factors including the viral load, the nature and quality of the specimen, and the sample‐handling technique. On one hand, a low Se (25.9%) and high Sp (98.4%) of the Ag‐RDT were shown in this study in comparison to RT‐PCR on Abbott m2000 in all suspected COVID‐19 cases. Although there is a good Sp of RDT found in this study compared to other studies, it should be noted that the Se was lower than that reported by Porte et al. and Randriamahazo et al., which was 93.9% and 62.66%, respectively [15, 16]. These divergent findings between the studies could be attributed to the use of 3 mL of VTM in our study and a buffer that could lead to a dilution of the antigen and, hence, a reduction in the Se of the Ag‐RDT [15]. Additionally, the Se of the STANDARD Q COVID‐19 test according to the nature of specimens was also low in suspected COVID‐19 cases with only nasopharyngeal swabs (26.32%) and those with nasopharyngeal samples (25.00%). This finding indicates that the performance of the STANDARD Q COVID‐19 assay seems to be better on nasopharyngeal specimens compared to nasopharyngeal/oropharyngeal specimens, although the overall diagnostic performance of Ag‐RDT is still inadequate for all specimen types in this study. This finding supports the recommendation of the manufacturer on the use of human nasopharyngeal swabs for screening of COVID‐19 [17].

Regarding the analytical performance of the test in our study, Ag‐RDT was not sensitive on the specimens of suspected COVID‐19 cases with Ct values ≥ 20, whereas its Se increased to 41.18% on specimens with Ct < 20. Other studies pointed out that the rapid antigen detection test is capable of detecting SARS‐CoV‐2 with high Se in nasopharyngeal specimens with a high viral load equivalent to at least 1.7 × 10^5^ copies/mL (Ct < 25), but the Se decreases significantly when the viral load is low, translating to Ct values above 30 [18].

Interestingly, our study showed fairly good sensitivities in confirmed COVID‐19 cases with cough (81.48%), asthenia (66.67%), fever/shivering (62.96%), shortness of breath (62.96%), and headache (62.96%). Randriamahazo et al. in 2021 reported in their study that RDT Se increased in highly symptomatic patients. This may be related to the fact that symptomatic patients have a higher viral load over a long period of time [16]. In addition, Porte et al. indicated in their study on the evaluation of a new rapid antigen‐based test for the diagnosis of SARS‐CoV‐2 in respiratory specimens in 2020 that antigenic tests might be more sensitive in the early phase of symptomatic infection on upper respiratory swabs [15].

Finally, the values of the Ag‐RDT were above 0.70, and Cohen′s kappa was between 0.21 and 0.39 demonstrating the usefulness in diagnosing COVID‐19 and its minimal agreement with the gold standard test (RT‐PCR). The diagnostic accuracy from this study correlates with the findings from the studies of Ouedraogo et al. in Burkina Faso and Mandal et al. in Nepal in 2022 who reported accuracy values of 85.82% and 78.9% [16], respectively. Regarding the concordance analysis (Cohen′s kappa) between Ag‐RDT and RT‐PCR, it varies from one study to another [19]. This could be attributed to the fact that Cohen′s *k* value is influenced by data distribution, the Se of diagnostic tests, the period of disease onset, and the presence of bias [19–21].

## 5. Limitations

The main limitations of this study were the unavailability of some clinical data such as the duration of the evolution of symptoms or the date of risky contacts and the absence of data on the quantification of viral load. This data could have provided some insight on the clinical performance of the Ag‐RDT in comparison to RT‐PCR in suspected COVID‐19 cases. Our sample size may also represent one of the limitations of this study due to the wide confidence intervals.

## 6. Conclusion

The study findings showed that the Ag‐RDT is useful in suspected COVID‐19 cases and the Se of the antigenic test using the STANDARD Q COVID‐19 Ag test was low with fair agreement in comparison to the molecular technique (RT‐PCR) using the Abbott m2000rt system. Although the Q COVID‐19 Ag‐RDT (SD BIOSENSOR, Republic of Korea) is an important tool for the early diagnosis of SARS‐CoV‐2, contributing to timely disease management in healthcare settings, its low Se requires that a quantitative molecular test should always be used when there is a strong suspicion of SARS‐CoV‐2 and the result of the Ag‐RDT is negative.

## Disclosure

All authors reviewed and approved the final manuscript for publication. Abdoul‐Salam Ouédraogo takes overall responsibility for the integrity of the work.

## Conflicts of Interest

The authors declare no conflicts of interest.

## Author Contributions

Abdoul‐Salam Ouédraogo and Yacouba Sawadogo designed the study. Abdoul‐Salam Ouédraogo, Yacouba Sawadogo, Herman Karim Sombie, Aicha Ilboudo, and Jessica Julie Chantal Samba designed the statistical analysis plan. Yacouba Sawadogo, Aicha Ilboudo, and Herman Karim Sombie contributed to the statistical analysis, interpretation of results, and drafting of the first version of the manuscript.

## Funding

No funding was received for this manuscript.

## Data Availability

The data that support the findings of this study are available from the corresponding author upon reasonable request.

## References

[1] Raharimanantsoa O. L. , Razakarivony F. A. , Andriamiadanalisoa A. O. , Rajaona R. A. , Rakotoarisoa R. , Randrianarimanana S. E. R. , and Raobela L. , Multiple infectious cranial nerve palsies in COVID-19, Journal Français D′ophtalmologie. (2020) 43, no. 10, e351–e353, 10.1016/j.jfo.2020.08.001, 33250085. PMC7543693 33250085

[2] Boushab B. M. , Fall-Malick F. Z. , Kelly M. , Ould Ahmed Baba E. , Ould Sidi Mohamed O. , Ould Baba S. W. , and al E. , Caractéristiques épidémio-cliniques, biologiques et radiologiques des adultes atteints de COVID-19 au centre hospitalier de Kiffa, Assaba (Mauritanie), Revue Malienne D′infectiologie et de Microbiologie. (2021) 16, no. 1, 25–31, 10.53597/remim.v16i1.1756.

[3] WHO , Coronavirus (COVID-19) Dashboard, https://data.who.int/dashboards/covid19/cases.

[4] Fourati S. , Audureau E. , Chevaliez S. , and Pawlotsky J. M. , Évaluation de la performance diagnostique des tests rapides d′orientation diagnostique antigéniques COVID-19, 2020, Laboratoire de Virologie et le Service de Santé Publique des Hôpitaux Universitaires Henri-Mondor (AP-HP, Université Paris-est-Créteil, et INSERM).

[5] WHO , Détection des antigènes à l′aide de tests immunologiques rapides pour le diagnostic de l′infection à SARS-CoV-2: orientations provisoires, Organisation Mondiale de la Santé. (2020) https://iris.who.int/server/api/core/bitstreams/6d1bea26-2853-4a97-9edb-21a60fed3b87/content.

[6] Henri Gautier O. , Serge Theophile S. , Gilles Ismael T. , Abdou Azaque Z. , Tegwinde Rebeca C. , Sylvie Z. , Tani S. , Charlemagne D. , Oumarou O. , Abdoul Rahamani N. , Charles S. , Sophie P. , Zakariya Y. , and Lassana S. , Assessment of Two Rapid Antigen Tests to Detect SARS-CoV-2 in Laboratory Setting in Ouagadougou, Burkina Faso, International Journal of Virology. (2023) 19, no. 1, 6–12, 10.3923/ijv.2023.6.12.

[7] Abdou Azaque Z. , Henri Gautier O. , Serge Théophile S. , Tegwinde Rebeca C. , Sylvie Z. , Oumarou O. , Dinanibè K. , Dezemon Z. , Charlemagne D. , Alidou K. , Charles S. , Zakariya Y. , Tani S. , and Lassana S. , Performance Assessment of STANDARD TM Q COVID-19 Ag Test in Ouagadougou Burkina Faso, Microbes and Infectious Diseases. (2023) 4, no. 3, 713–723.

[8] Serge Théophile S. , Abdou Azaque Z. , Gilles Ismael T. , Rebeca Tegwindé C. , Dinanibè K. , Oumarou O. , Tani S. , Charlemagne D. , Abdoul Rahamani N. , Charles S. , Sophie P. , Zakariya Y. , Lassana S. , and Henri Gautier O. , Performance of STANDARDTM Q COVID-19 Ag and STANDARDTM F COVID-19 Ag FIA Compared to Genes Detected by RT-PCR in Burkina Faso, Science et Technique, Sciences de la Santé. (2024) 2, no. 2, 61–79.

[9] Jacob S. and Martin R. , Diagnostic Testing Accuracy: Sensitivity, Specificity, Predictive Values and Likelihood Ratios, 2020, https://europepmc.org/article/nbk/nbk557491. 32491423

[10] Gutiérrez G. , Gresh L. , Pérez M. , Elizondo D. , Avilés W. , Kuan G. , Balmaseda A. , and Harris E. , Evaluation of the Diagnostic Utility of the Traditional and Revised WHO Dengue Case Definitions, PLoS Neglected Tropical Diseases. (2013) 7, no. 8, 10.1371/journal.pntd.0002385, 2-s2.0-84883353341. PMC3749970 23991237

[11] McHugh M. L. , Interrater Reliability: The Kappa Statistic, Biochemia Medica. (2012) 22, no. 3, 276–282, 10.11613/BM.2012.031, 23092060. 23092060 PMC3900052

[12] Samaké D. , Coulibaly M. , Kéita M. , Guindo O. , Dembélé M. , Traoré A. , Kané A. M. , Traoré A. S. , Konaté M. D. , and Traoré B. K. , La COVID-19 à Mopti: Aspects épidémiologique, clinique, thérapeutique et évolutif, Revue Malienne D′infectiologie et de Microbiologie. (2021) 16, no. 1, 47–51, 10.53597/remim.v16i1.1760.

[13] Savadogo M. , Ouattara A. , Dahani C. K. , Nikiéma O. , Traoré S. , Nagréongo B. , and Sawadogo N. , Profil épidémiologique et clinique des cas suspects de COVID-19 reçus au CHU Yalgado Ouédraogo du Burkina Faso, Revue Malienne D′infectiologie et de Microbiologie. (2021) 16, no. 1, 7–10, 10.53597/remim.v16i1.1753.

[14] Donamou J. , Bangoura A. , Camara L. M. , Camara D. , Traoré D. A. , Abékan R. J. M. , Sossa L. K. , Mohamed C. M. , Abdoulaye T. , Yalla C. A. , Atigou D. A. , Saliou S. M. , and Baele P. , Caractéristiques épidémiologiques et cliniques des patients COVID-19 admis en réanimation à l′hôpital Donka de Conakry, Guinée: étude descriptive des 140 premiers cas hospitalisés, Anesthésie & Réanimation. (2021) 7, no. 2, 102–109, 10.1016/j.anrea.2021.01.001.

[15] Porte L. , Legarraga P. , Vollrath V. , Aguilera X. , Munita J. M. , Araos R. , Pizarro G. , Vial P. , Iruretagoyena M. , Dittrich S. , and Weitzel T. , Evaluation of a Novel Antigen-Based Rapid Detection Test for the Diagnosis of SARS-CoV-2 in Respiratory Samples, International Journal of Infectious Diseases: IJID: Official Publication of the International Society for Infectious Diseases. (2020) 99, 328–333, 10.1016/j.ijid.2020.05.098, 32497809. 32497809 PMC7263236

[16] Randriamahazo T. R. , Andrianarivelo A. M. , Rakotoarivo A. T. , Raheritiana T. M. , Rakotovao L. A. , Randriamanantany Z. A. , Rakoto Alson A. O. , and Rasamindrakotroka A. , Evaluation of Antigen-Based Rapid Detection Test for the Diagnosis of SARS CoV-2 in Low-Income Countries, Journal of Virological Methods. (2022) 300, 114409, 10.1016/j.jviromet.2021.114409. 34896454 PMC8654736

[17] WHO , IFU for STANDARD Q COVID-19 Ag Test (EUL 0563-117-00), 2020, WHO.

[18] Scohy A. , Anantharajah A. , Bodéus M. , Kabamba-Mukadi B. , Verroken A. , and Rodriguez-Villalobos H. , Low Performance of Rapid Antigen Detection Test as Frontline Testing for COVID-19 Diagnosis, Journal of Clinical Virology: The Official Publication of the Pan American Society for Clinical Virology. (2020) 129, 104455, 10.1016/j.jcv.2020.104455. 32485618 PMC7240272

[19] Ouedraogo H. G. , Zoure A. A. , Sagna T. , Soubeiga S. T. , Compaoré T. R. , Zingue D. , Zida S. , Dabiré C. , Kagambega A. , Sawadogo C. , Yabre Z. , and Sangare L. , Performance of the PanbioTM COVID-19 Ag Rapid Test in a Health Care Setting in Ouagadougou, Burkina Faso, African Journal of Microbiology Research. (2022) 16, no. 11, 334–342, 10.5897/AJMR2022.9651.

[20] Byrt T. , Bishop J. , and Carlin J. , Bias, Prevalence and Kappa, Journal of Clinical Epidemiology. (1993) 46, no. 5, 423–429, 10.1016/0895-4356(93)90018-V, 2-s2.0-0027210597, 8501467. 8501467

[21] Sim J. and Wright C. C. , The Kappa Statistic in Reliability Studies: Use, Interpretation, and Sample Size Requirements, Physical Therapy. (2005) 85, no. 3, 257–268, 10.1093/ptj/85.3.257, 15733050. 15733050

